# Influence of Porosity Gradient Distribution on Mechanical and Biological Properties of Gyroid-Based Zn-2Mg Scaffolds for Bone Tissue Engineering

**DOI:** 10.3390/ma18184399

**Published:** 2025-09-21

**Authors:** Shuxin Chen, Kai Liao, Youwen Yang, Huiming Chen, Renkai Huang

**Affiliations:** 1 School of Mechanical and Electrical Engineering, Jiangxi University of Science and Technology, Ganzhou 341000, China; 2 State Key Laboratory of Multiphase Flow in Power Engineering, Xi’an Jiaotong University, Xi’an 710049, China

**Keywords:** Gyroid porous bone scaffolds, gradient porosity, finite element analysis, mechanical properties, biological properties

## Abstract

To address the insufficient matching between high strength and low elastic modulus in traditional metal bone scaffolds and the issue of secondary surgical removal, this study used degradable zinc magnesium alloy as the material to study the relationship between porosity gradient distribution and mechanical and biological properties of Gyroid porous bone scaffolds. We established three groups of scaffolds with different porosity gradient distribution, including uniform, axial gradient, and radial gradient. Numerical simulation experiments were conducted for axial compression. The simulation results show that compared to uniform and axial gradients, radial gradient scaffolds have the highest Young’s modulus and exhibit exceptional load-bearing capacity. The results of sample compression experiments show that under the same (average) porosity, the elastic modulus of uniform porous scaffolds and radial gradient porous scaffolds was not significantly different, but reverse radial gradient scaffolds exhibited superior yield strength relative to uniform porous scaffolds. Moreover, forward radial gradient scaffold extracts showed lower toxicity on the in vitro proliferation of mouse calvarial pre-osteoblast cells. By designing a forward radial gradient Gyroid porous bone scaffold, it is expected to obtain a biodegradable Zn-2Mg porous bone scaffold with excellent mechanical and biological properties.

## 1. Introduction

As the core pillar of human biomechanics and fundamental building blocks for life activities, bones play vital roles in supporting the body, protecting internal organs, enabling movement, facilitating hematopoiesis, and storing minerals, making them essential organs [1,2]. However, bone injury cases caused by accidents, surgeries, or tumor resections have been increasing annually in recent years [3]. When bone defects exceed 4 mm, relying solely on autografts or allografts becomes insufficient for effective repair [4]. The primary challenges include limited donor bone supply, severe shortage of transplantable bone tissue, growing supply-demand imbalance, and post-implantation risks of immune rejection [5]. To address these issues, humanity has embarked on a long journey of developing artificial bone substitutes. From traditional metal scaffolds to modern biodegradable alternatives, the evolution of artificial bone materials has been extensive. As early as the 17th century, bone grafting primarily used allogeneic bone. While this method offered simple acquisition and good osseointegration, it frequently triggered immune rejection after implantation. By the 19th century, autografts and allogeneic bone were applied in bone transplantation, yet still faced challenges like limited donor availability, immune rejection, and postoperative complications [6,7].

In the early 20th century, titanium (pure and alloyed), Co-Cr alloys and other metallic biomaterials emerged as primary candidates for artificial bone scaffolds, initially being applied in clinical orthopedic procedures [8]. Extensive clinical trials demonstrated that these metal scaffolds exhibited exceptional biocompatibility, corrosion resistance, and mechanical properties [9]. For example, titanium alloys were renowned for their excellent biocompatibility, coupled with their high mechanical properties, qualifying titanium alloy as a reliable metal option for biological scaffolds [10,11]. However, it had been observed that the main drawback of using solid metals as bone implants was that the Young’s modulus of conventional bulk implants (e.g., Ti: 110 GPa; Co-Cr alloys: 220–230 GPa; SS316L: 193 GPa) significantly exceeds that of bone (10–30 GPa), leading to mechanical incompatibility. The disparity in equivalent stiffness between the implant and surrounding tissue can lead to a physical phenomenon of stress shielding [12]. This abnormal mechanical environment suppresses osteoblast activity while accelerating osteoclast-mediated bone resorption, ultimately resulting in osteoporosis and bone loss.

Entering the 21st century, people have discovered biodegradable materials for medical use. This material is a biocompatible material that can be gradually broken down into small molecules and metabolized in the human body through enzymatic hydrolysis, hydrolysis, or microbial action. Medical degradable metallic materials primarily include iron (Fe)-based alloys, magnesium (Mg)-based alloys, and zinc (Zn)-based alloys [13]. However, the development of bone scaffold materials still faces numerous challenges and unresolved issues. Porous structure design can be used to decrease the elastic modulus and alleviate stress shielding, while the strength of porous implants decreases significantly with increasing porosity, which may lead to fracture failure shortly after implantation [14]. The most urgent issue at present is the mismatch between low modulus and high strength [15]. How to design a bone scaffold that can adapt to the elastic modulus of human bones and has high load capacity is the core challenge for the clinical application of biodegradable bone scaffolds and bone implants.

The Gyroid porous structure, first discovered in 1970 by NASA scientist Alan Schoen, was originally designed to develop lightweight yet high-strength new materials [16,17]. It is a typical three period minimal surface (TPMS) in which the surface exhibits zero curvature at all points, thereby minimizing surface tension. Composed of interconnected channels, it forms a complex labyrinthine morphology [18,19,20]. As a novel artificial bone structure in orthopedics, it closely mimics human skeletal architecture, demonstrating tremendous potential for clinical bone trauma repair [21]. Compared with other types of TPMS, Gyroid porous structures demonstrate superior manufacturability, mechanical robustness, and osteogenic potential [22]. The main reason for the excellent proliferation and growth of bone cells is high specific surface area and permeability of Gyroid porous structures, which are beneficial for bone cell adhesion and nutrient transport, effectively promoting bone tissue regeneration and repair [22,23]. In addition, the use of gradient porous structure design based on Gyroid can achieve the regulation of the biological and mechanical properties of implants, and optimize the biological performance under the constraint of elastic modulus and bone matching [24].

Gyroid porous bone scaffolds feature high specific surface area and permeability [17], which facilitate the attachment, growth, adhesion, migration, and proliferation of osteoblasts. These characteristics endow the scaffold with high biological activity, effectively promoting bone tissue regeneration and repair.

When designing Gyroid porous bone scaffolds, material properties are a critical factor influencing their mechanical performance. The specific selection of materials directly affects the scaffold’s strength, stiffness, and toughness—mechanical characteristics that ultimately determine its effectiveness in biological applications. On the one hand, the elastic modulus serves as a key indicator for evaluating mechanical strength. Materials with a higher elastic modulus demonstrate greater resistance to deformation under external forces, making them more durable in clinical settings. On the other hand, the elastic modulus of the bone scaffold cannot be above the desired range, otherwise stress shielding could take place. The reasonable range of its elastic modulus is 10–30 GPa [12]. Zinc (Zn), with its excellent biodegradability and biocompatibility, is a promising material for bone repair. However, pure zinc exhibits relatively low mechanical strength when used in load-bearing applications. Alloying elements induce substantial strengthening effects in pure zinc.

Compared with other biomedical materials, the characteristics and advantages of Zn-2Mg zinc alloy are as follows: (1) Its elastic modulus (45 ± 11.616 GPa) [25] is close to that of human bones (10–30 GPa), that can effectively decrease the stress shielding effect. In addition, its yield strength (210 ± 8.878 MPa) [25] far exceeds the yield strength of human bone (133.6 ± 34.1 MPa) [26], which can meet the needs of daily human movement load-bearing. (2) It has excellent biocompatibility, does not release any gas when degraded, and has no cytotoxicity. Zinc plays a crucial role as a trace element in the human body, which can promote the growth of bone cells [27]. Moreover, Zn exhibits excellent antibacterial properties and osteogenic potential, making it widely recognized as a promising bone implant material [28,29]. Based on the above analysis, this study sets the material of the bone scaffold as Zn-2Mg. However, there is still a stress shielding problem with Zn-2Mg bone scaffolds, which can be solved by designing porous structures. However, it faces the challenge of unclear relationship between porosity gradient distribution and mechanical properties.

Therefore, this study designed three sets of Gyroid porous bone scaffold models, including uniform, axial gradient, and radial gradient porous bone scaffolds, to study the influence of different porosity gradient distributions on the mechanical properties of porous bone scaffolds, making the performance of artificial bone scaffolds closer to the mechanical properties of human bones, improving stress shielding problems, and enhancing load-bearing capacity.

## 2. Materials and Methods

### 2.1. Design of Gyroid-Based Porous Structures

**Figure 1:**
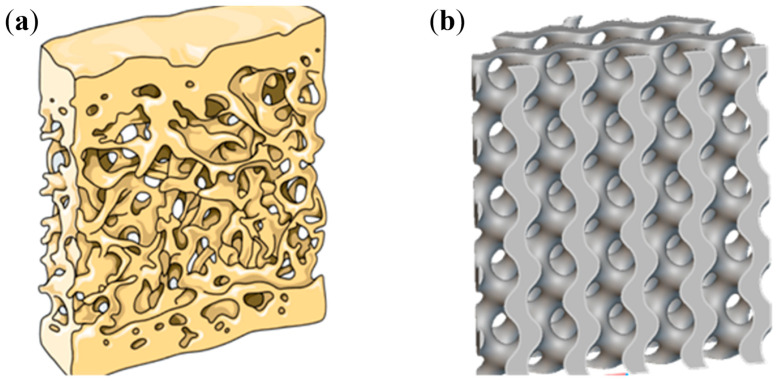
Design of biomimetic bone scaffolds based on Gyroid porous structures: (**a**) Human bone scaffold structures; (**b**) Gyroid-based porous scaffold structures.

This article adopts the design of a biomimetic bone scaffold based on the porous structure of Gyroid, as shown in Figure 1. The standard function expression for this structure is as follows:

(1) sin(X)cos(Y)+sin(Y)cos(Z)+sin(Z)cos(X)=t

where the variable *t* represents the offset parameter. By adjusting this constant, different cell structures with varying wall thicknesses can be obtained, thus forming the design expression for the structure.

By scaling its period, we define *X* = 2*πx/l*, *Y* = 2*πy/l*, and *Z* = 2*πz/l*, where *l* denotes the unit cell size of the crystal. This transformation effectively scales the period to l, resulting in a repeating unit size of l along the x, y, and z axes. Consequently, Equation (2) is derived, which serves as the design expression for this study.

(2) $$\sin\frac{2\pi x}{l}\cos\frac{2\pi y}{l}+\sin\frac{2\pi y}{l}\cos\frac{2\pi z}{l}+\sin\frac{2\pi z}{l}\cos\frac{2\pi x}{l}=\frac{2\pi t}{l}$$

(3) $$d=\frac{2\pi t}{l}$$

**Figure 2:**
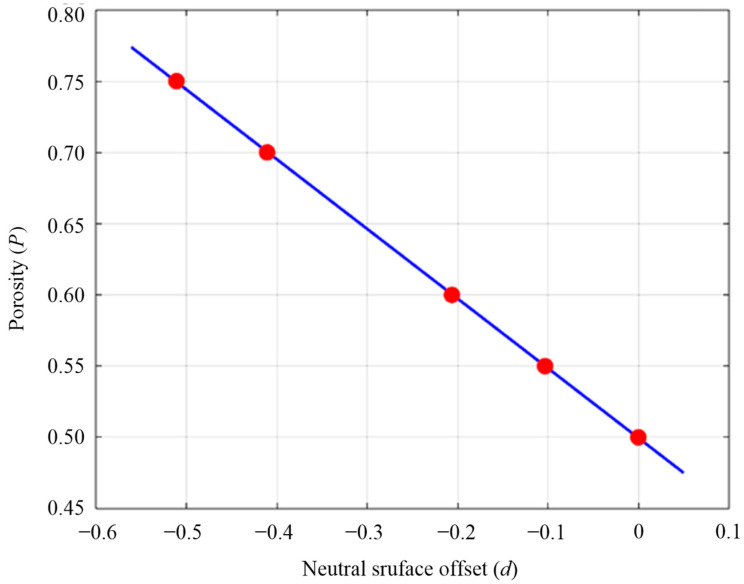
Fit results of neutral surface offset value and porosity (SD = 0.003).

In Equation (3), *d* represents the critical parameter, neutral surface offset (mm), for controlling wall thickness in the modeling software nTopology 5.9.2 used in this study. By adjusting its magnitude, scaffolds for bone tissue engineering can be designed with different porosities. Porous structures based on the gyroid unit cell was designed using nTopology software, with porosities of 50%, 55%, 60%, 65%, 70%, and 75% (corresponding neutral plane offset parameters are shown in Table 1). To obtain the mathematical relationship between parameter *d* and porosity (*P*), MATLAB R2021a software was used to fit the data of Table 1. The fit result was presented in Figure 2, revealing a highly linear relationship between the two parameters.

At the same time, the fitting equation is obtained from Figure 2, as follows:

(4) P=−0.49865d+0.4995

The fitting function has a standard deviation (SD) of 0.003. All porous bone scaffolds presented in this study were designed using a Gyroid structure with a 4 mm unit cell size. For a Gyroid unit with a cell size of l = 4 mm, the functional relationship between porosity and offset parameter t calculated according to Equations (2) and (4) is as follows:

*P* = (0.311 *t* + 0.5)% (5)

The pore size determines the mechanical strength of bone scaffolds. Although smaller pores can meet strength requirements, they will reduce permeability and impair the efficiency of transporting nutrients within the bone. This also impedes osteoblast growth and vascular infiltration. Conversely, excessive pores, though beneficial for cell proliferation and nutrient delivery, weaken mechanical integrity. Such compromised strength fails to withstand physiological loads, ultimately compromising scaffold stability and hindering bone tissue healing.

### 2.2. Modeling of Porous Bone Scaffolds of Uniform and Gradient Porosity

We established three groups of Gyroid porous bone scaffold models, including uniform, axial gradient, and radial gradient scaffolds measuring 20 mm in diameter, 20 mm in height, and a unit cell size of 4 mm. In addition, for the convenience of finite element analysis, 1 mm rigid plates were set up above and below the model.

**Figure 3:**
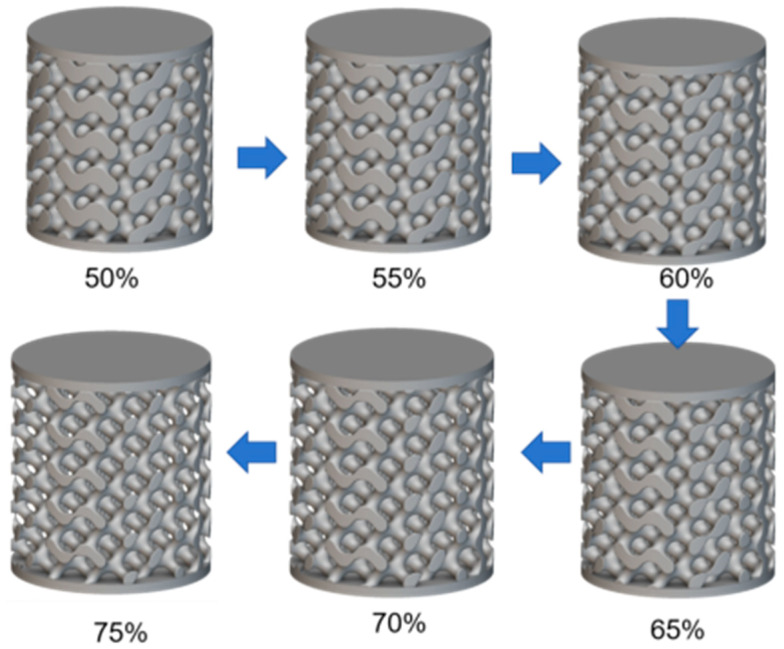
Homogeneous porosity bone scaffold models.

First, six uniform cylindrical bone scaffold models were designed according to the previous Table 1, with porosity of 50%, 55%, 60%, 65%, 70% and 75%, respectively, as shown in Figure 3.

**Figure 4:**
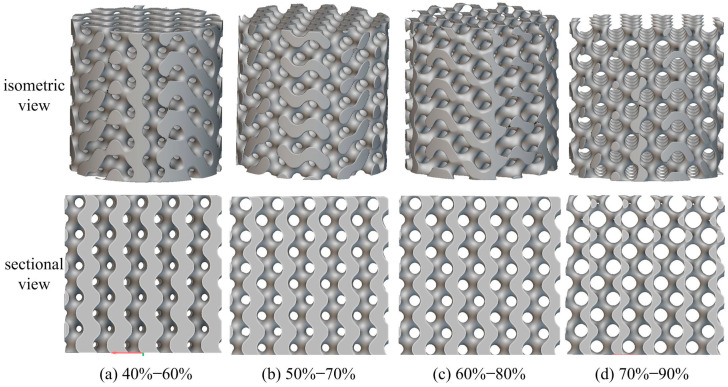
Axial gradient variation porous bone scaffold models (The top and bottom rigid plates are hidden).

Then, by introducing gradient function change instructions (Scalar Field: Axis) in nTopology, porosity changes along the *Z*-axis direction were achieved to design four sets of axial gradient porous bone scaffold models with 20% gradient changes. As shown in Figure 4, the first one shows a variation in porosity from the bottom 40% along the *Z*-axis to the top 60%, exhibiting 50% average porosity in the bone scaffold. The fourth one shows a variation in porosity from the bottom 70% along the *Z*-axis to the top 90%, with an average porosity of 80% for the bone scaffold.

**Figure 5:**
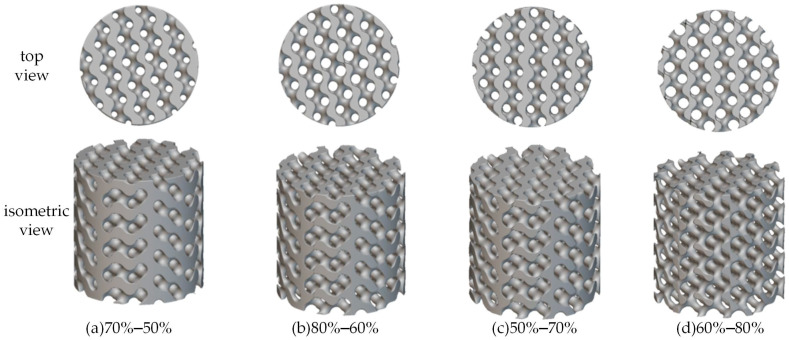
(**a**,**b**) are reverse radial gradient models, where porosity decreases from the center to the edge. (**c**,**d**) are forward radial gradient models, where porosity increases from the center to the edge (The top and bottom rigid plates are hidden).

Finally, this study developed 10 radial gradient porous scaffold models with a 20% porosity gradient variation, including 5 scaffolds with forward gradients (30–50%, 40–60%, 50–70%, 60–80%, and 70–90%) and 5 scaffolds with reverse gradients (50–30%, 60–40%, 70–50%, 80–60%, and 90–70%). In addition, uniform gradient bone scaffolds were established as reference groups based on the average porosity of radial gradient porous bone scaffolds, respectively. Two types of radial gradient porous bone scaffolds with reverse gradients of 70–50% (porosity decreases radially from 70% in the center to 50%), 80–60% (porosity decreases radially from 80% in the center to 60%), and two forward gradients of 50–70% (porosity increases radially from 50% in the center to 70%), 60–80% (porosity increases radially from 50% in the center to 70%) are illustrated in Figure 5a–d, respectively.

### 2.3. Finite Element Analysis of Porous Bone Scaffolds

This study used Ansys 2022R1 software to perform static mechanics analysis on the bone scaffold model. In the FEA of statics mechanics, the porous scaffold material was set as Zn-2Mg. The specific properties of materials (Zn-2Mg) used in FEA are listed in Table 2. Firstly, tetrahedral elements with a default size were used to mesh models. Then, boundary conditions were defined, and a load was applied. The top and bottom rigid plates had an axial compression displacement of 2 mm applied and were fixed, respectively. Under compressive loading conditions, we evaluated the compressive stress, strain, and failure load of the scaffold to determine the mechanical stability of porous bone scaffold models with different porosities or pore gradients under compressive forces.

The equivalent Young’s modulus (E) was selected as the mechanical evaluation metric for porous bone scaffolds, calculated from the linear region slope of the stress–strain curve. Its numerical representation of the bearing characteristics of the material during the elastic deformation stage directly affects the stress distribution state at the scaffold–bone tissue interface. A marked disparity between scaffold and bone elastic modulus initiates stress shielding, consequently inducing bone resorption. The equivalent Young’s modulus can be calculated from equivalent stress and strain using Hooke’s law, and its formula is given as follows:

(6) E=σϵ=FA∆LL

where *σ* represents the equivalent stress during deformation, calculated as *σ = F/A*, where *F* denotes the pressure applied during deformation and *A* is the area of the stressed plate. ϵ indicates strain, representing the degree of material deformation, calculated as *ϵ = ΔL/L*_0_, where Δ*L* is the displacement of the bone scaffold and *L*_0_ is its total height (20 mm). *E* stands for the equivalent Young’s modulus, reflecting material stiffness, which is the ratio of the stress magnitude (*σ*) corresponding to strain (*ϵ*).

### 2.4. Sample Manufacturing and Mechanical Testing

We selected 6 uniform gradient scaffolds (50%, 55%, 60%, 65%, 70%, 75%), 2 axial gradient scaffolds (50–70%, 60–80%), and 8 radial gradient scaffolds (forward 40–60%, 50–70%, 60–80%, 70–90%; reverse 60–40%, 70–50%, 80–60%, 90–70%) for sample manufacturing using laser powder bed melting (LPBF). The samples were built with specific process parameters (30 μm layer thickness, 70 W laser power, 1300 mm/s scanning speed, 0.07 mm scanning spacing, 100 μm spot size) in an argon gas-filled, IPG fiber laser equipped LPBF machine (BLT S210; Xi’an Bright Laser Technologies Co., Ltd., Xi’an, China). Zinc alloy Zn-2Mg, with spherical morphology and particle size range of 15–53 μm, was used to build the samples.

Uniaxial compression testing was performed using a CMT5105 universal testing machine (SANS, Shenzhen, China) under ambient conditions, with each sample tested in duplicate. The upper load cell was driven downward at 2 mm/min, with a total compression displacement of 6 mm. Stress–strain curves were derived from recorded force-displacement data, with the elastic modulus calculated as the slope of the linear region.

### 2.5. In Vitro Cell Culture

The mouse calvarial pre-osteoblast cell line MC3T3-E1 was selected for in vitro evaluation of LPBF-fabricated Zn-2Mg uniform and gradient porous scaffolds. This cell line was provided by the Cell Bank of the Chinese Academy of Sciences (Shanghai, China). Cells were cultured in alpha-MEM (Gibco, New York, NY, USA) supplemented with 10% fetal bovine serum (FBS), 100 U/mL penicillin, and 100 μg/mL streptomycin. LPBF fabricated Zn-2Mg porous scaffolds, including uniform porous scaffolds with 60% porosity and gradient porous scaffolds with optimal mechanical performance and average porosity close to 60%, were cultured in the same medium for 24 h to prepare extracts in humidified atmosphere (5% CO_2_, 37 °C). The supernatant was collected for cell viability test via LIVE/DEAD staining.

MC3T3-E1 cells were initially plated at 5000 cells/cm^2^ in 12-well plates and maintained in alpha-MEM for 24 h to establish cell–substrate attachment. After that, the cell culture media in each well were substituted by scaffold extracts. For the control group, cells were cultured in alpha-MEM medium without any additives in empty wells. Cells were incubated at 37 °C in a 5% CO_2_ humidified atmosphere for 1 or 7 days. Then, cells were stained with Calcein-AM and Propidium iodide (Invitrogen, Carlsbad, CA, USA) for 15 min after phosphate-buffered solution (PBS) washing. Finally, observation was performed with a Leica TCS SP5 confocal microscope (Wetzlar, Germany) following cell fixation on glass slides.

## 3. Results and Discussion

### 3.1. Finite Element Analysis (FEA)

#### 3.1.1. Uniform Porous Bone Scaffolds

**Figure 6:**
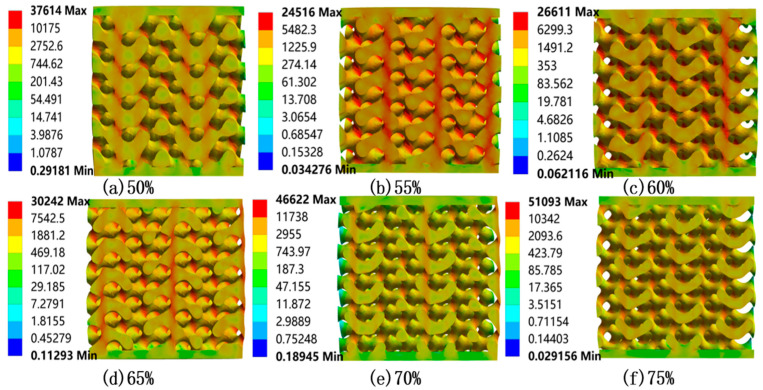
Equivalent stress contours of uniform porous bone scaffolds (Cross-sectional views, MPa).

**Figure 7:**
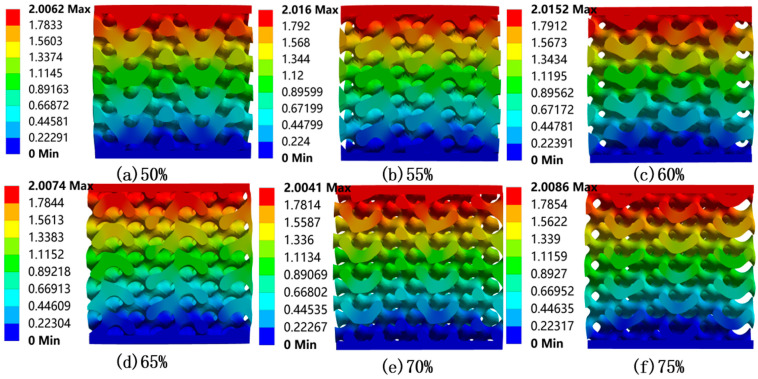
Displacement distribution of uniform porous bone scaffolds (Cross-sectional views, mm).

The equivalent stress contours of six uniform bone scaffold models are shown in Figure 6, and the total deformation distribution is shown in Figure 7. Based on the results of Figure 6 and Figure 7, we obtain the following findings:

(1) As a typical TPMS, the stress distribution of the Gyroid structure exhibits the characteristics of “global dispersion and local concentration”. With the continuous transition of the surface, stress can be uniformly transmitted. When subjected to axial displacement load, the surface with continuous curvature will disperse the external load in multiple directions, thus forming a global stress “dilution” effect.

(2) However, in areas where there is a sudden change in geometry, such as the contact surfaces at both ends of a cylinder or the transition of curvature, it is easier to form local stress peaks due to excessively high local curvature. From the equivalent stress section view, it can be seen that the high stress areas in orange and red are concentrated at the corners of the structure, while the low stress areas in dark blue are distributed in areas with relatively gentle internal curvature.

To reduce the risk of stress concentration, gradient design (such as radial or axial porosity gradient) can be considered to improve the uniformity and biocompatibility of stress distribution through smooth transitions.

**Figure 8:**
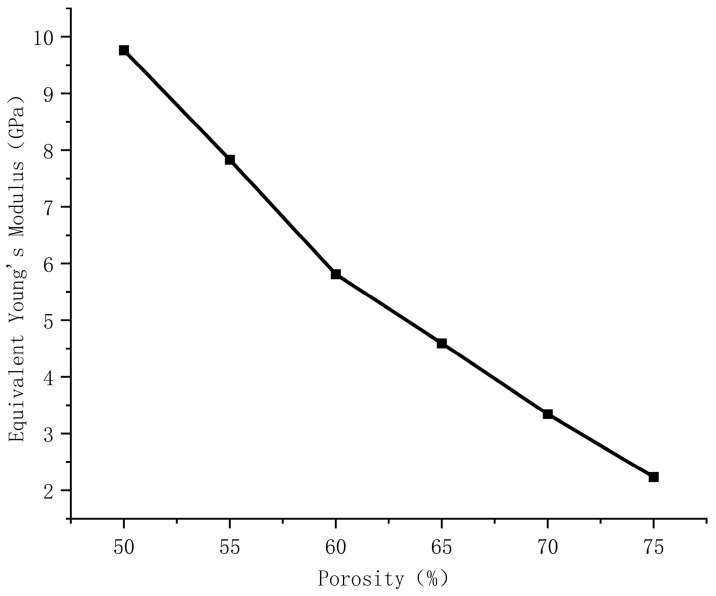
Equivalent Young’s modulus of uniform porous bone scaffolds with different porosity.

Based on the FEA results of uniform porous scaffolds with varying porosities, Table 3 presents the Young’s modulus values calculated from the stress–strain curve slopes during the elastic deformation phase. Fit the porosity of the uniform porous scaffold with the corresponding equivalent Young’s modulus value in MATLAB software to quantify the relationship between the two, as shown in Figure 8. The results indicate that under the same displacement load, as the porosity of the bone scaffold increases, its equivalent Young’s modulus will decrease. According to Table 3, as the porosity increases from 50% to 75%, the equivalent Young’s modulus of the bone scaffold reduces from 9.763 GPa to 3.233 GPa, with a cumulative decrease of 66.89%. When the porosity is ≤60%, for every 5% increase in porosity, the equivalent Young’s modulus decreases by a maximum of 2.02 GPa. When the porosity is greater than 60%, the decrease decreases, and for every 5% increase in porosity, the equivalent Young’s modulus decreases by at least 1.112 GPa.

#### 3.1.2. Axial Gradient Porous Bone Scaffolds

**Figure 9:**
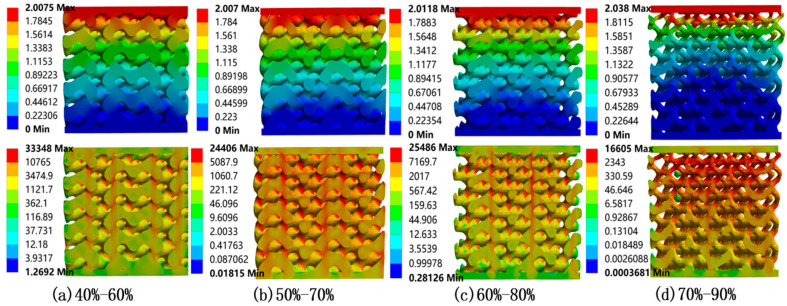
Displacement distribution (the image above, mm) and equivalent stress contours (the image below, MPa) of axial gradient porous bone scaffolds.

Similarly, static compression simulations were conducted on four axial gradient models, and their displacement distributions and equivalent stress contours were presented in Figure 9. The simulation results show that the stress peak (red area) was mainly concentrated in the upper half, and the stress gradually decreases with decreasing height. This is because the porosity of the top surface is larger than that of the bottom surface, and the ability of the high porosity part to withstand deformation is weaker than that of the low porosity part.

**Figure 10:**
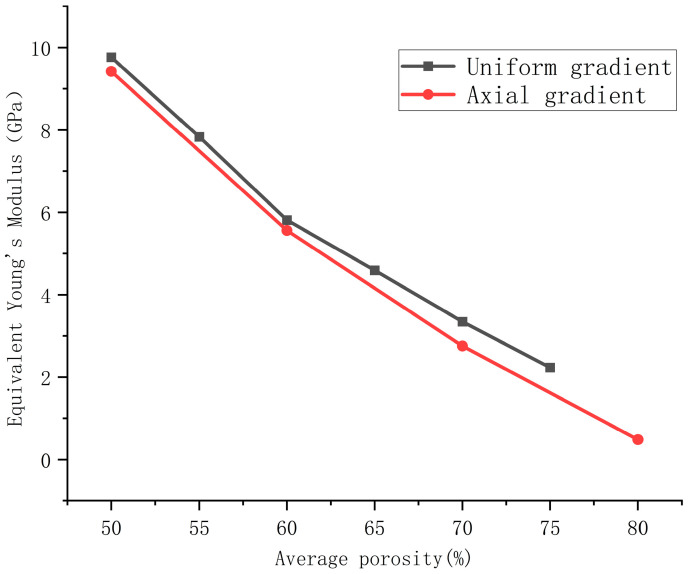
Comparison of elastic modulus between axial gradient and uniform porous bone scaffolds.

The average porosity of the four axial gradient porous bone scaffolds is 50%, 60%, 70%, and 80%, respectively, and their Young’s moduli are shown in Table 4. In addition, we also compared the elastic modulus of the axial gradient porous structure with the uniform porous bone scaffold corresponding to its average porosity, as shown in Figure 10.

We can clearly see from Figure 6, Figure 9 and Figure 10 that porosity and porosity gradient exert a substantial influence on the mechanical characteristics of bone scaffolds. The mechanical properties of axial gradient porous bone scaffolds are slightly worse than those of uniform porous bone scaffolds corresponding to their average porosity. Taking a bone scaffold with an axial gradient distribution of porosity of 50–70% (average porosity of 60%) as an example, its equivalent Young’s modulus is 5.560 GPa. However, the equivalent Young’s modulus of a uniform porous bone scaffold with a porosity of 60% can reach 5.812 GPa. Similarly, comparing the 60–80% axial gradient bone scaffold with a uniform porous bone scaffold with a porosity of 70%, the Young’s moduli are 2.757.1 GPa and 3.345 GPa, respectively. From the trend of experimental data, this suggests that as the porosity of the scaffold continues to increase, the equivalent Young’s modulus of both gradient and uniformly distributed scaffolds show a decreasing trend.

#### 3.1.3. Radial Gradient Porous Bone Scaffolds

**Figure 11:**
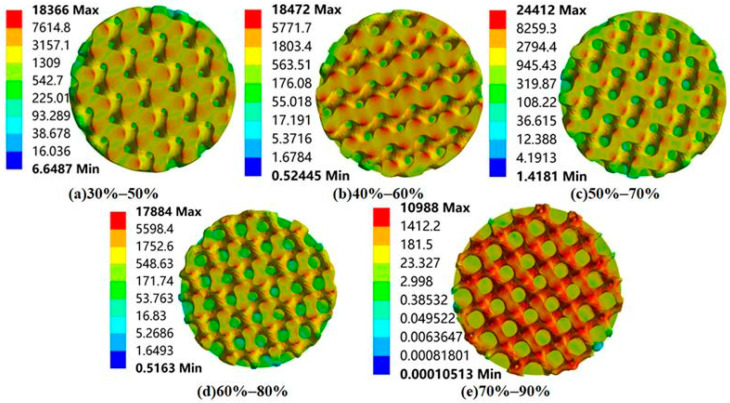
Equivalent stress contours of forward radial gradient porous bone scaffolds (vertical view, MPa).

**Figure 12:**
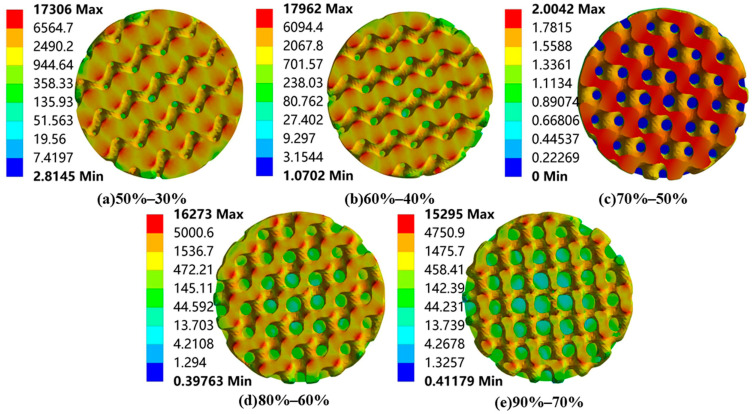
Equivalent stress contours of reverse radial gradient porous bone scaffolds (vertical view, MPa).

Finally, static compression finite element analysis was performed on the radial gradient porous bone scaffold to obtain the total deformation, equivalent strain, and equivalent stress distribution. Based on the comprehensive consideration of the stress gradient characteristics and structural symmetry of the radial gradient scaffold, the bone scaffold is horizontally cut to obtain a top sectional view. The equivalent stress profile of the forward gradient scaffold is shown in Figure 11, and the equivalent stress profile of the reverse gradient scaffold is shown in Figure 12.

According to Figure 11 and Figure 12, it can be seen that different radial gradient pores significantly alter the mechanical properties of bone scaffolds. In the forward gradient scaffolds, due to the high porosity at the edges, high-stress zones occupy a restricted area, and the stress peak is mainly concentrated in the axial part. On the other hand, the reverse gradient scaffold is exactly the opposite, with lower axial porosity, smaller areas of high stress, and stress peaks mainly concentrated at the circumferential edges.

The forward radial gradient Gyroid porous bone scaffold has a porosity that gradually decreases from the outer surface of the scaffold towards the center of the circle (for example, the porosity of the outer layer is 60% and that of the inner layer is 40%), forming a characteristic of external softness and internal rigidity. This structural characteristic can be understood in practical applications as: the high porosity of the outer layer is prone to elastic deformation, which can absorb the impact of external forces. Reducing the surface stiffness of a bone scaffold better matches the elastic modulus of natural bone, which minimizes the stress shielding effect post-implantation. At the same time, the relatively lower porosity of the inner layer can provide high strength support for the stent, ensuring that the stent has certain mechanical properties and making the main structure of the stent less prone to deformation.

The porosity of the reverse radial Gyroid porous bone scaffold gradually decreases from the center of the scaffold to the outer surface (for example, the porosity of the inner layer is 60%, while that of the inner layer is 40%), forming the characteristics of internal softness and external rigidity. Due to this unique porosity characteristic, in practical applications, the low porosity of the inner layer can provide a wider growth environment for bone cells and a continuous load transfer path, avoiding local stress concentration in the scaffold caused by random pore distribution in uniform gradient structures. The low porosity structure of the outer layer can guarantee that the bone scaffold remains rigid and does not deform when subjected to external impact.

**Figure 13:**
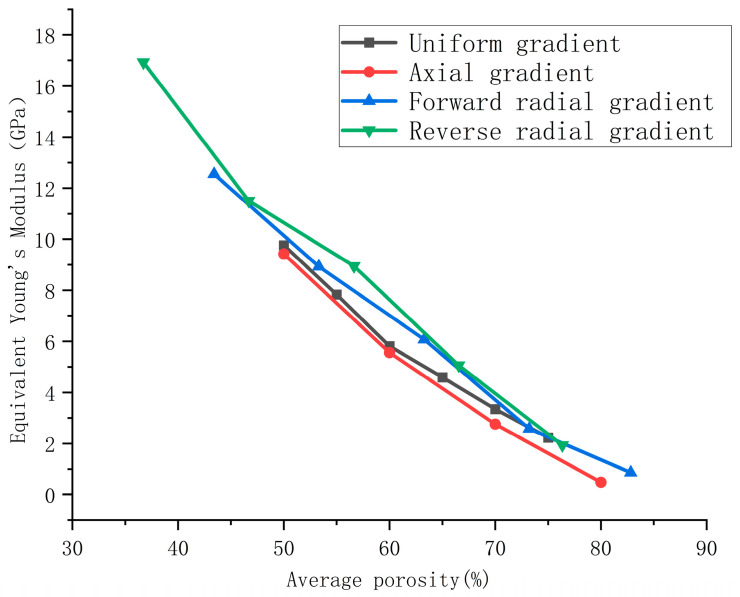
Comparison of equivalent Young’s modulus of porous scaffolds with different porosity and porosity gradient distributions.

Equivalent Young’s modulus data from each experimental group were compiled. The equivalent Young’s modulus values for the forward radial gradient groups are shown in Table 5, while those for the reverse radial gradient groups are presented in Table 6. Simultaneously, experimental data from four groups—uniform gradient, axial gradient, forward radial gradient, and reverse radial gradient—were fitted using MATLAB software, as illustrated in Figure 13.

Equivalent Young’s modulus of the radial gradient scaffold models was obtained through finite element method, where equivalent Young’s modulus of forward radial gradient scaffolds was provided in Table 5 and that of the reverse radial gradient scaffolds was shown in Table 6.

Based on the above analysis, the correlation between the elastic modulus and average porosity of four sets of scaffolds with uniform gradient, axial gradient, forward radial gradient, and reverse radial gradient was plotted using MATLAB software. The results are shown in Figure 13. From Figure 13, it can be seen that under the same average porosity conditions, the order of mechanical performance is as follows: the reverse radial gradient scaffold is optimal, followed by the forward radial gradient scaffold, then the uniform scaffold, and the axial gradient scaffold is the worst.

### 3.2. Manufacturability and Mechanical Properties

**Figure 14:**
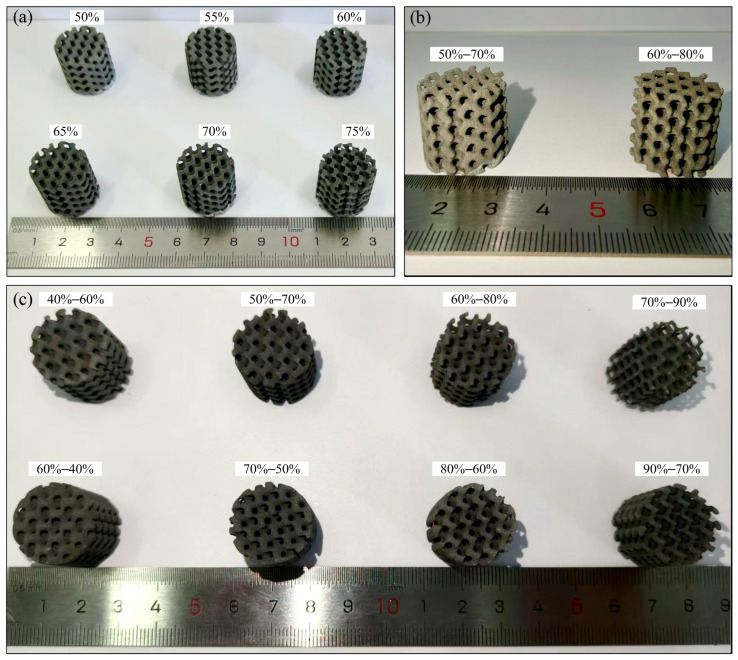
The LPBF as-built Zn-2Mg porous samples: (**a**) uniform porous scaffolds; (**b**) axial gradient porous scaffolds; (**c**) radial gradient porous scaffolds.

**Figure 15:**
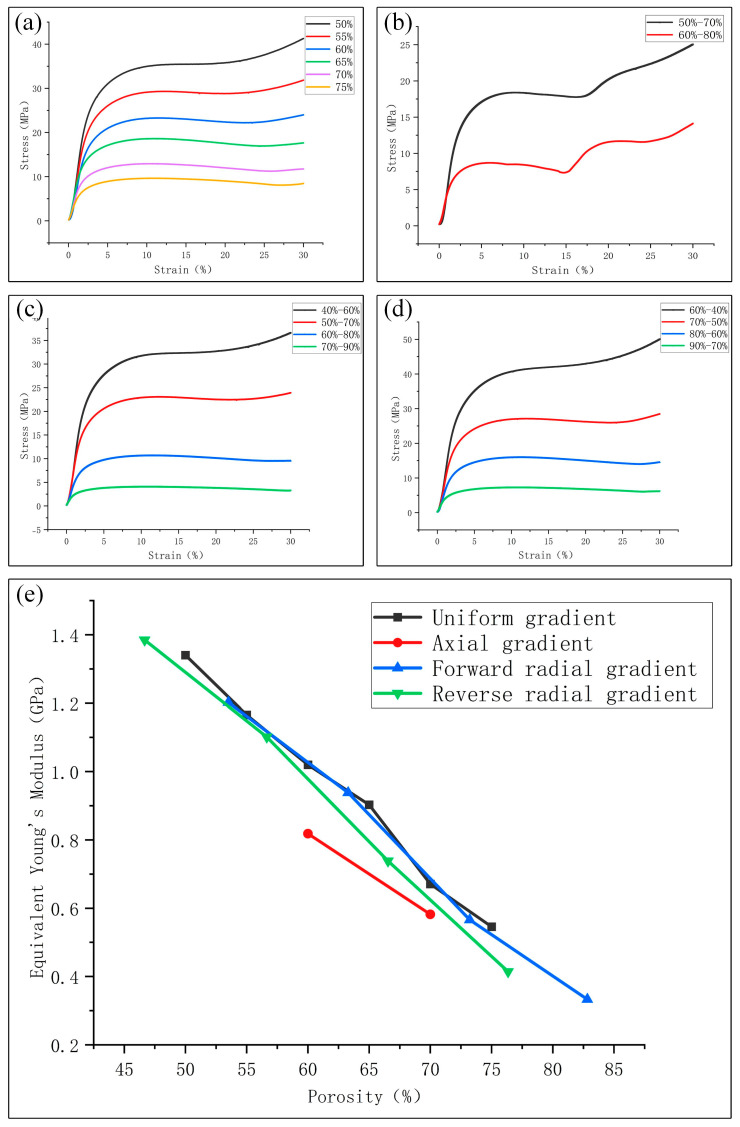
Compressive engineering stress–strain curves of Zn-2Mg porous scaffolds with different (average) porosity and equivalent elastic modulus of Zn-2Mg porous scaffolds with different porosity distributions and average porosities: (**a**) Uniform porous scaffolds; (**b**) Axial gradient porous scaffolds; (**c**) Forward radial gradient porous scaffolds; (**d**) Reverse radial gradient porous scaffolds; (**e**) Comparison of elastic modulus of Zn-2Mg porous scaffolds with different porosity gradient distributions.

The experimental samples prepared for compression tests are presented in Figure 14. Figure 15a–d show the compression stress–strain curves of uniform and gradient scaffolds manufactured by LPBF with different (average) porosities, respectively. Curves demonstrate the typical three-stage characteristics of all porous scaffolds, including initial elastic deformation, plastic deformation, and densification stage. As is well known, the elastic modulus is related to the slope characterizing the material’s initial linear elastic response. Therefore, the elastic modulus values of LPBF fabricated scaffolds with different (average) porosity and porosity distributions can be directly compared through stress–strain curves. It is evident that the elastic modulus reduces with the increase in (average) porosity. In addition, Figure 15e reveals that compared with uniform scaffolds with the same porosity, the axial gradient scaffold has a lower elastic modulus than the uniform scaffold (consistent with the FEA results of Figure 13), and the radial gradient scaffold exhibits similar compressive elastic modulus (the finite element simulation in Figure 13 shows that the elastic modulus of the radial gradient scaffold is slightly greater than that of the uniform scaffold), but the compressive yield strength is higher (especially the radial gradient scaffolds), suggesting that the radial gradient scaffold’s design improves mechanical performance.

Notably, the elastic modulus values of porous scaffolds obtained through sample compression experiments were generally smaller than those obtained using finite element methods. This is because the finite element analysis uses an ideal model, and LPBF manufactured samples have defects such as internal pores, surface roughness, and dimensional deviations.

### 3.3. In Vitro Biocompatibility

**Figure 16:**
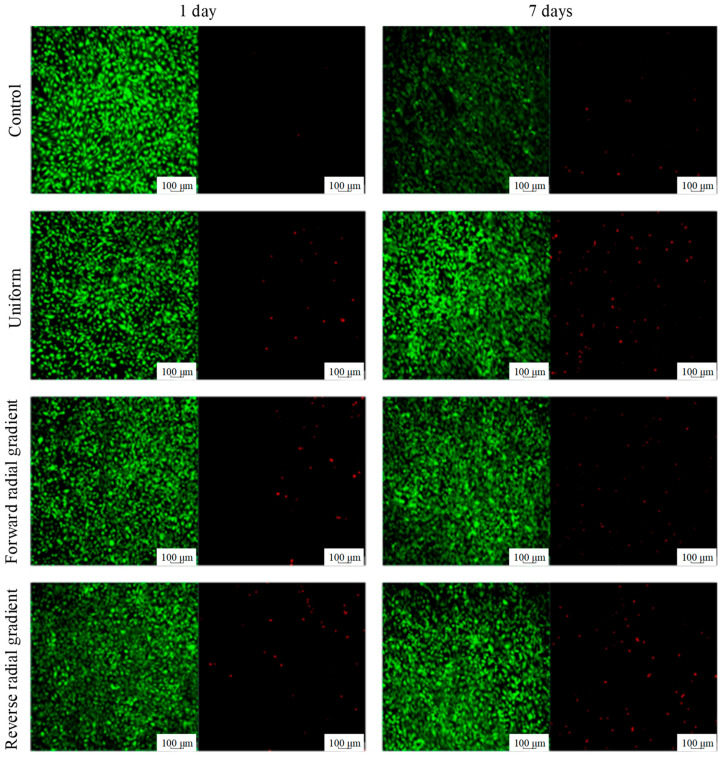
Laser scanning confocal microscope images of cells cultured in extracts for different periods.

Based on FEA and compression test results, it can be concluded that the radial gradient porous scaffold has the best mechanical properties under the same porosity. Therefore, forward radial gradient scaffolds (50–70%) with an average porosity close to 60%, reverse radial gradient scaffolds (70–50%) with an average porosity close to 60%, and uniform scaffolds with a porosity of 60% were selected as the experimental groups, and α-MEM medium was used as the control group. Laser scanning confocal microscope images of MC3T3-E1 cells cultured in extracts of LPBF fabricated Zn-2Mg porous scaffolds for different period were illustrated in Figure 16.

Green and red staining identified live and dead cells, respectively. A considerable number of dead cells were present in all test groups, but the control group had the least number of dead cells, followed by the forward radial gradient group. After 1 day of cultivation, all MC3T3-E1 cells in the test group showed a classic fusiform shape with a diameter of approximately 20–30 microns, indicating normal cell growth. After 7 days, MC3T3-E1 cells cultured in the extract showed abundant pseudopods, releasing an extracellular matrix and starting to extend toward nearby cells, indicating good cell diffusion and proliferation. In addition, as the cell incubate time increased from 1 day to 7 days, the number of live cells in all test groups significantly increased. These results indicate that the extracts of LPBF fabricated Zn-2Mg porous scaffolds were beneficial for cell proliferation. Notably, the densities of dead cells in the forward radial gradient scaffold group were significantly lower than that in the uniform scaffold group and the reverse radial gradient scaffold group, demonstrating that the forward radial gradient scaffold resulted in improved cytocompatibility.

Good biocompatibility is essential for the use of biomaterials in orthopedics. The extracts of LPBF fabricated Zn-2Mg porous scaffolds were used to simulate the microenvironment after implantation. MC3T3-E1 cells showed good survival rate when cultured in extracts of Zn-2Mg porous scaffolds, indicating that the Zn-2Mg porous scaffold manufactured by LPBF has good cell compatibility. As an essential nutrient for the human body, magnesium activates many enzymes and promotes protein synthesis. Therefore, it can be reasonably inferred that Zn-2Mg releasing Mg^2+^ may help enhance cell proliferation and adhesion, thereby accelerating bone healing during implantation. Of even more significance, the in vitro cell experiments also showed that cells cultured in extracts of the forward radial gradient scaffolds grew better than in those of uniform scaffolds and reverse radial gradient scaffolds. The enhanced biocompatibility of the forward radial gradient scaffolds may be due to its structural characteristic of “large outer and small inner” pore size, which slows down the diffusion of toxic substances produced in the small inner pore area.

## 4. Conclusions

This study aims to investigate how a porosity gradient affects the mechanical and biological properties of Gyroid-structured porous bone scaffolds. Uniform, axial gradient, and radial gradient bone scaffold models were established in nTopology. Finite element analysis, samples manufacturing and in vitro cell culture experiments were conducted using Zn-2Mg as the bone scaffold material. The key findings are as follows:

(1) The finite element analysis results indicate that the equivalent Young’s modulus of the radial gradient scaffold is the maximum under the same average porosity. Its mechanical properties are the most excellent among all gradient porous structures. In addition, the mechanical properties of the reverse radial gradient scaffold were better than those of the forward radial gradient scaffold. Compared to uniform scaffolds and axial gradient scaffolds, porous bone scaffolds with radial porosity gradients can optimize load-bearing capacity and alleviate edge stress shielding, making them more suitable as bone repair materials for load-bearing implants.

(2) Due to the inevitable internal defects in the porous scaffold manufactured by LPBF, including pores, surface roughness, dimensional deviations, etc., the elastic modulus of the porous scaffold obtained from the sample compression experiment was smaller than the finite element analysis results. Unlike finite element simulation, under the same (average) porosity, the elastic modulus of radial gradient porous scaffolds was not significantly different from that of uniform porous scaffolds. However, the trend of scaffolds with different porosity gradient distributions gradually increasing with porosity was similar to the results of finite element analysis.

(3) In vivo cell culture experiments have found that the Zn-2Mg porous scaffolds based on Gyroid have good biocompatibility. In addition, MC3T3-E1 cells cultured with forward radial gradient porous scaffold extracts showed the lowest cell mortality rate. Taking into account both mechanical and biological properties, the forward radial gradient porous structure is suitable for the design of Zn-2Mg bone scaffolds.

## Figures and Tables

**Table 1:** The correspondence between porosity and neutral surface offset parameters.

Porosity (*P*)	50%	55%	60%	65%	70%	75%
Neutral surface offset parameter (*d*)	−0.0004	−0.10356	−0.2065	−0.3065	−0.4108	−0.511

**Table 2:** Characteristics of Zinc Alloy in Finite Element Analysis.

Material	Elastic Modulus/GPa	Poisson’s Ratio
Zn-2Mg	45	0.3

**Table 3:** Analysis table of uniform gradient porosity results.

Porosity	50%	55%	60%	65%	70%	75%
Isoelastic Young’s Modulus (GPa)	9.763	7.832	5.812	4.594	3.345	2.233

**Table 4:** Average porosity and equivalent Young’s modulus of axial gradient porous bone scaffolds.

Porosity (Average Porosity)	40–60% (50%)	50–70% (60%)	60–80% (70%)	70–90% (80%)
Equivalent Young’s Modulus (GPa)	9.422	5.560	2.757	0.483

**Table 5:** Equivalent Young’s modulus of forward radial gradient porous bone scaffolds.

Porosity (Average porosity)	30–50% (43.41%)	40–60% (53.31%)	50–70% (63.29%)	60–80% (73.22%)	70–90% (82.82%)
Equivalent Young’s modulus (GPa)	13.15	8.63	6.18	2.38	0.8

**Table 6:** Equivalent Young’s modulus of reverse radial gradient porous bone scaffolds.

Porosity (Average porosity)	50–30% (36.72%)	60–40% (46.67%)	70–50% (56.64%)	80–60% (66.54%)	90–70% (76.35%)
Equivalent Young’s modulus (GPa)	16.93	11.50	8.95	4.062	1.735

## Data Availability

The original contributions presented in the study are included in the article. Further inquiries can be directed to the corresponding authors.
